# Design and fabrication of a metastable β-type titanium alloy with ultralow elastic modulus and high strength

**DOI:** 10.1038/srep14688

**Published:** 2015-10-05

**Authors:** Shun Guo, Qingkun Meng, Xinqing Zhao, Qiuming Wei, Huibin Xu

**Affiliations:** 1 School of Materials Science and Engineering, Beihang University, Beijing 100191, P. R. China; 2 Institute for Advanced Materials, Jiangsu University, Zhenjiang 212013, P. R. China; 3 Department of Mechanical Engineering and Engineering Science, University of North Carolina at Charlotte, 9201 University City Blvd., Charlotte NC 28223-0001, USA

## Abstract

Titanium and its alloys have become the most attractive implant materials due to their high corrosion resistance, excellent biocompatibility and relatively low elastic modulus. However, the current Ti materials used for implant applications exhibit much higher Young’s modulus (50 ~ 120 GPa) than human bone (~30 GPa). This large mismatch in the elastic modulus between implant and human bone can lead to so-called “stress shielding effect” and eventual implant failure. Therefore, the development of β-type Ti alloys with modulus comparable to that of human bone has become an ever more pressing subject in the area of advanced biomedical materials. In this study, an attempt was made to produce a bone-compatible metastable β-type Ti alloy. By alloying and thermo-mechanical treatment, a metastable β-type Ti-33Nb-4Sn (wt. %) alloy with ultralow Young’s modulus (36 GPa, versus ~30 GPa for human bone) and high ultimate strength (853 MPa) was fabricated. We believe that this method can be applied to developing advanced metastable β-type titanium alloys for implant applications. Also, this approach can shed light on design and development of novel β-type titanium alloys with large elastic limit due to their high strength and low elastic modulus.

Titanium and titanium alloys have become the most attractive implant materials owing to their lightweight, high corrosion resistance, excellent biocompatibility, high specific strength and relatively low elastic modulus^1^,^2^,^3^. Among the mechanical properties essential for implants, elastic modulus is of paramount importance whose value should be as close as possible to that of natural human bone, since mismatch in modulus between the implant and bone can lead to “stress shielding effect” and eventually to implant failure^1^,^4^,^5^. However, conventional Ti materials used for implant applications, such as pure Ti and the most popular (α + β) type Ti-6Al-4V, have much higher Young’s modulus (~110 GPa) than human bone (~30 GPa)^1^. Furthermore, V and Al ions released from Ti-6Al-4V have been held responsible for long-term health problems including Alzheimer disease, neuropathy and osteomalacia^6^,^7^. Therefore, in the past few decades, extensive efforts have been undertaken to develop non-cytotoxic metastable β-type Ti alloys with low elastic modulus. Up to date, β-type Ti alloys developed for implant application can have Young’s modulus in the range of 50 ~ 80 GPa^1^,^8^,^9^,^10^, still not low enough to match that of human bone. Therefore, the development of β-type Ti alloys with modulus comparable to that of human bone has become an ever more pressing subject in the area of advanced biomedical materials.

Previous experimental and computational results have consistently shown that from the perspective of modulus design, the total amount of β-stabilizers in the metastable β-type Ti alloys should be reduced as low as possible, because the Young’s modulus of β-phase decreases with decrease in the total content of β-stabilizers (e.g. Nb, Ta, etc.)^11^,^12^,^13^,^14^. However, insufficient β-stabilizers results in the thermoelastic β→α″ (martensite) transformation upon quenching or deformation at room temperature, leading to remarkable decrease in yield strength of the metastable β-Ti alloy^15^.

Recent experiments show that in addition to alloying, grain refinement and high density of dislocations introduced by thermo-mechanical treatment can suppress the β→α″ martensitic transformation^16^,^17^,^18^. This suggests that with the assistance of thermo-mechanical treatment, β-phase with less β-stabilizers can survive at room temperature. Obviously, this points to the looming hope of developing β-type Ti alloys with elastic modulus approaching that of human bone.

In addition to bone-compatible Young’s modulus, decent yield strength is yet another essential metric of Ti alloys used for implants to accommodate the complex stress state, including tension, compression and torsion, which may be experienced by the implants during routine activities^1^,^8^. In general, however, high strength and low elastic modulus seem to be inter-exclusive for solid materials, particularly for metals and alloys. Indeed, most of the biomedical β-type Ti alloys with low modulus usually exhibit much lower yield strength than conventional Ti-6Al-4V^1^,^4^,^8^. Although these alloys can be strengthened by precipitation hardening through aging or annealing treatment, their Young’s moduli will be inevitably and undesirably increased^1^,^9^,^10^. In this case, the “stress shielding” problem caused by the high modulus of the implant will be even exacerbated. As such, in order to improve the performance of biomedical β-type Ti alloys as implant materials, it is indispensable to develop metastable β-type Ti alloys with concurrently ultralow modulus and high strength.

Recently, Matsumoto and Hanada *et al.* reported that upon appropriate thermo-mechanical processing the Ti-Nb-Sn alloy system can possess lower elastic modulus than other β-type Ti alloy systems, such as Ti-Nb^1^,^3^ and Ti-Nb-Zr-Ta^3^, and thus has a great potential for biomedical applications^17^,^19^. A typical composition of Ti-Nb-Sn system, Ti-33.6Nb-4Sn (wt. %), exhibits an elastic modulus of 40 GPa in the α“-martensitic state, after a series of thermo-mechanical treatments including groove rolling, swaging and cyclic tensile deformation^19^. This level of Young’s modulus in α“-martensitic Ti-33.6Nb-4Sn alloy is lower than that of existing β-type Ti alloys. Matsumoto *et al.* attributed this low modulus to the stabilization of α″-martensite, instead of the survival of metastable β phase^19^. Upon a reverse martensitic transformation from α″ to β upon heating, the β-phase Ti-33.6Nb-4Sn alloy was found to have a combination of low modulus (52 GPa) and high tensile strength (over 800 MPa)^17^. The results of Hanada and Matsumoto *et al.* reveal that the Ti-Nb-Sn alloy shows an obvious superiority in terms of low modulus. As such an ultralow modulus comparable to that of human bone might be obtained by appropriate compositional design and thermo-mechanical treatment in the Ti-Nb-Sn alloy system.

In this study, an attempt was made to produce a bone-compatible metastable β-type Ti alloy in the Ti-Nb-Sn alloy system. We combined compositional design and thermo-mechanical treatment (cold rolling plus annealing, designated as CRA henceforth) to fabricate a metastable β-type Ti-Nb-Sn alloy with high strength and ultralow modulus comparable to that of human bone. We believe that this method can be applied to developing novel metastable β-type titanium alloys for implant applications. Also, we envision that this approach can shed light on design and development of β-type titanium alloys with large elastic limit due to their high strength and low elastic modulus.

## Results and Discussion

**Figure 1:**
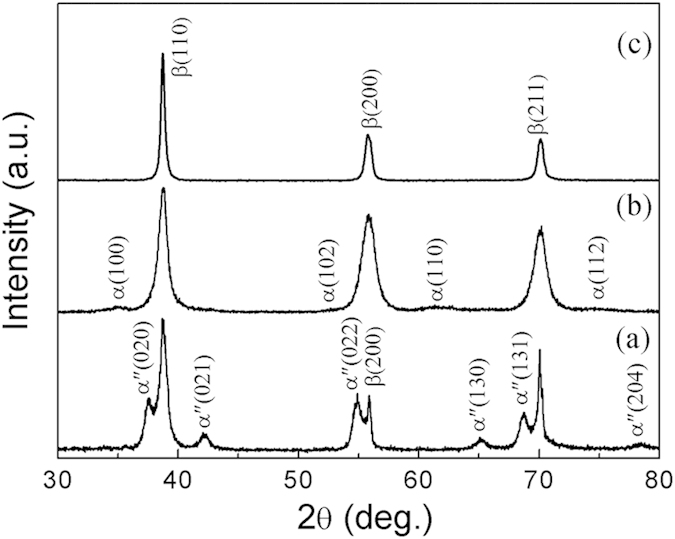
Room-temperature XRD results for solution treated (ST) (**a**), cold rolled plus annealed (CRA) (**b**) Ti-33Nb-4Sn specimens, and XRD results obtained at 473 K for the ST Ti-33Nb-4Sn specimen (**c**). Notice the appearance (**a**) and absence (**b**) of α″-martensite in the ST and CRA specimens, respectively. Evidence for α-phase can be identified in (**b**). Only β-peaks are present at 473 K (**c**).

The room-temperature XRD results for solution-treated (designated as ST henceforth) and cold-rolled plus annealed (CRA) Ti-33Nb-4Sn specimens are presented in Fig. 1a,b, respectively. Figure 1a provides unambiguous evidence for the parent β-phase, and the α″-martensite after solution treatment at 1073 K for 1 hour followed by quenching into water. The occurrence of β→α″ martensitic transformation on quenching can be attributed to the low content of β-stabilizers in the alloy. Interestingly, upon CRA at 673 K for 20 minutes, the α″ phase vanishes in the XRD profile, as shown in Fig. 1b. This suggests that α″-martensite formed before annealing is transformed back to β-phase upon annealing, and on subsequent cooling the martensitic transformation was suppressed. Instead, α-precipitates can be observed in the CRA specimen, as substantiated by the weak yet clear α-(100) and α-(110) peaks. Figure 1c shows the XRD results recorded at 473 K for the ST specimen of the same alloy. In comparison to its room-temperature counterpart (Fig. 1a), no diffraction peaks from α″-martensite can be observed, underscoring the notion that the β→α″-martensite transformation is reversible.

**Figure 2:**
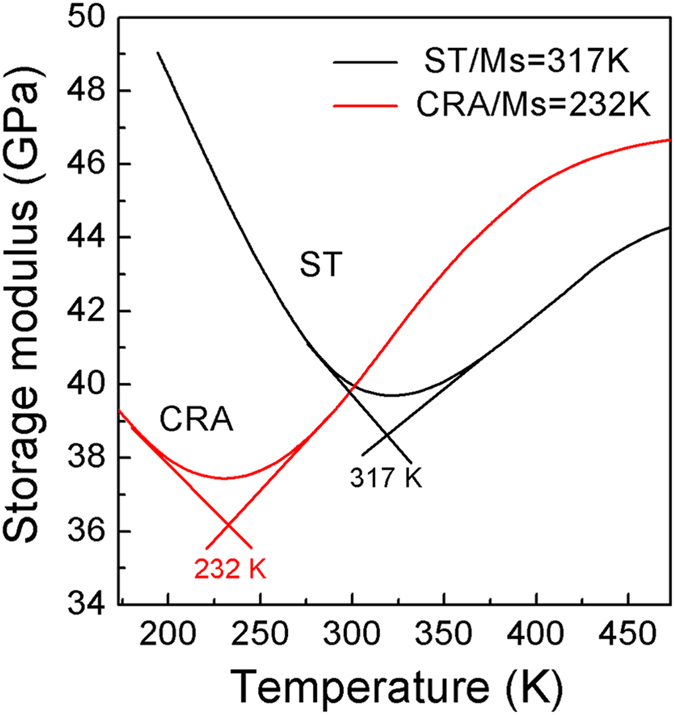
Storage modulus versus temperature during cooling for the solution treated (ST) and cold rolled plus annealed (CRA) Ti-33Nb-4Sn specimens, respectively.

The storage modulus, which can be utilized to characterize the Ms temperature from β to α″, was measured by dynamic mechanical analyzer (DMA) during cooling for the ST and CRA specimens, and the results are shown in Fig. 2. It is observed that the storage modulus of both ST and CRA Ti-33Nb-4Sn specimens first decreases with decreasing temperature and then increases with further decrease in temperature. According to Fig. 2, the martensitic transformation start (Ms) temperatures of the ST and CRA specimens are estimated to be 317 and 232 K, respectively. Note that the Ms temperature of the CRA Ti-33Nb-4Sn specimen is ~85 K lower than that of the ST specimen, suggesting that the β to α″ martensitic transformation can be effectively retarded or even suppressed by cold rolling and subsequent annealing, in line with the XRD results.

**Figure 3:**
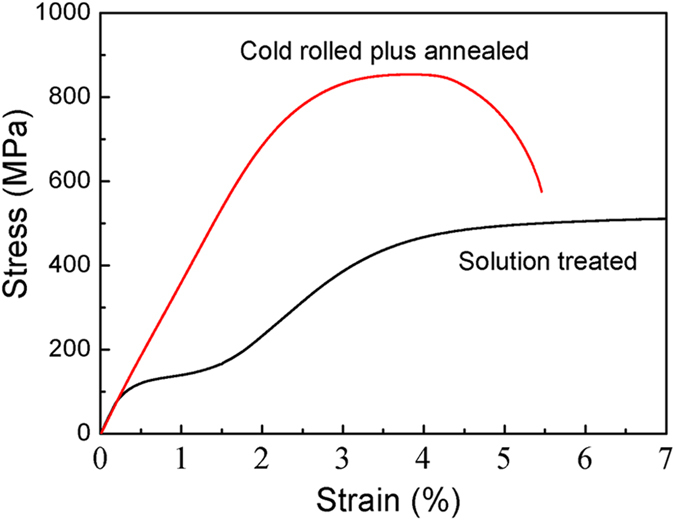
Representative tensile stress-strain curves of solution treated (ST) and cold rolled plus annealed (CRA) Ti-33Nb-4Sn specimens. Double yielding can be observed from the ST specimen where the first yielding is associated with a very low yield stress and can be attributed to stress-induced β→α″ martensitic transformation. The second yielding at a higher stress signals the plastic deformation of the material. The CRA specimen exhibits both very high yield and ultimate strengths. Slope of the elastic portion gives a Young’s modulus of only ~36 GPa, very close to that of human bone (~30 GPa).

Figure 3 gives the representative tensile stress-strain curves of ST and CRA Ti-33Nb-4Sn specimens. The ST specimen exhibits notable “double yielding” behavior. Obviously, the first yielding at ~107 MPa can be attributed to the stress-induced β→α″ martensitic transformation and the reorientation of the martensite variants; the second yielding is associated with the initiation of plastic deformation. Apparently, the alloy in such a condition is not a good candidate as implant material because of its relatively low yield (107 MPa) and ultimate tensile strength (509 MPa), although it does exhibit low Young’s modulus. However, with CRA treatment, the alloy is significantly strengthened, with a yield strength in the order of ~763 MPa and ultimate tensile strength of ~853 MPa. On top of this impressive strength level, we should note that the CRA specimen also exhibits an ultralow value of Young’s modulus of ~36 GPa. To the best of our knowledge, this level of Young’s modulus is the lowest ever recorded for a solid and bulk titanium alloy, and is much lower than those of the currently available biomedical Ti alloys^1^,^2^,^4^,^9^,^10^,^20^,^21^,^22^. What is more, it is only ~6 GPa higher than that of human bone.

Here, an important point to note is that the present Ti-33Nb-4Sn alloy can be further strengthened by raising annealing temperature or extending annealing time. For example, upon annealing at 723 K for 4 h, a high tensile strength of 1104 MPa can be obtained due to the massive formation of α precipitates, at the expensive of an increased Young’s modulus (see Fig. S2 in the supplementary information). Clearly, in comparison to high strength, low Young’s modulus is more important for implant alloys, since high Young’s modulus tends to exacerbate the “stress shielding” problem. Thus, in the present study, short-time annealing at low annealing temperature, i.e. annealing at 673 K for 20 minutes, was employed to limit the volume fraction of α-phase so as to obtain ultralow Young’s modulus.

**Figure 4:**
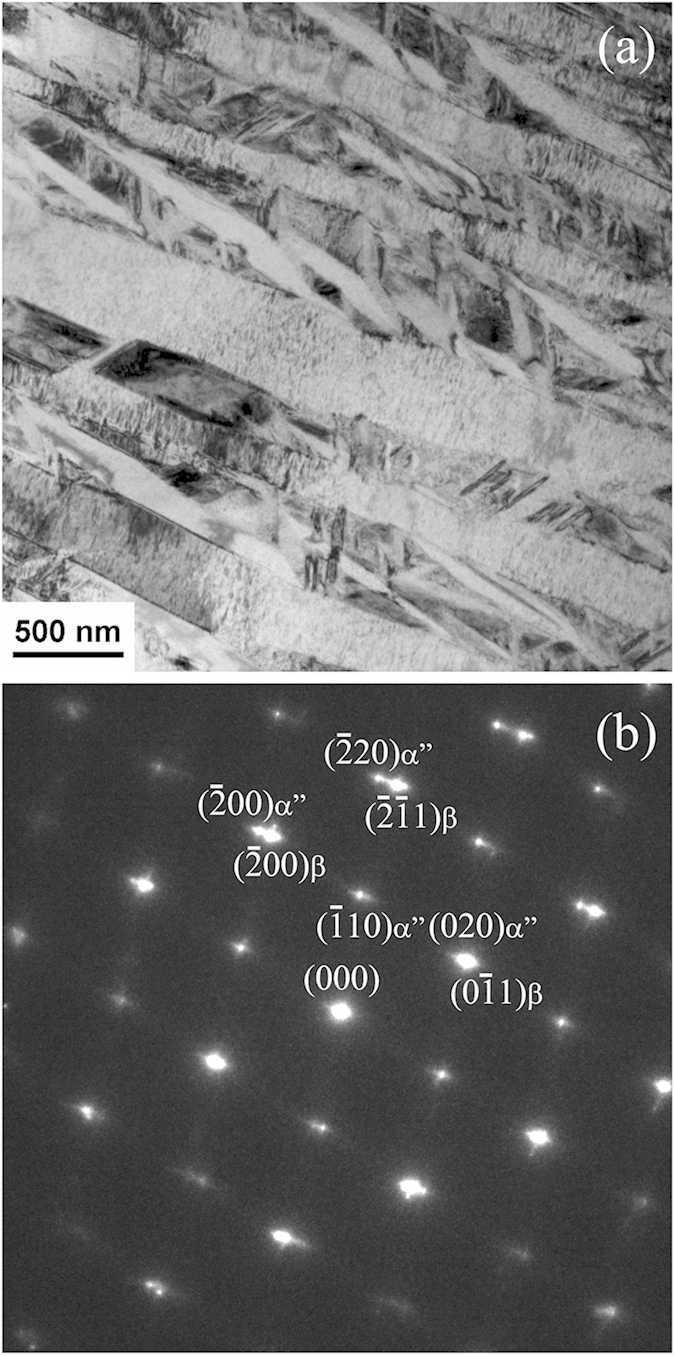
Bright field TEM image of solution-treated (ST) Ti-33Nb-4Sn specimen (**a**) and the corresponding electron diffraction pattern (**b**). The bright field image displays typical martensite structure. The indexed diffraction pattern suggests presence of α″-martensite within the β-matrix, in line with XRD results (Fig. 1(a)).

In order to uncover the mechanisms responsible for the much sought-after mechanical properties exhibited by the CRA specimens, we performed detailed TEM analyses of both the ST and CRA specimens. Figure 4a,b show a typical bright-field TEM image and the corresponding selected area electron diffraction (SAD) pattern of the ST specimen. In the bright-field image (Fig. 4a), lath-shaped α″-martensite can be observed within the β-phase matrix. The presence of α″-martensite can be further confirmed from the [110] β zone axis SAD pattern of Fig. 4b, where the reflections near the 1/2 positions indicate the existence of α″-martensite. It should be noticed that in this pattern, no reflection from ω-phase can be identified at 1/3 and 2/3 positions, suggesting no occurrence of the athermal ω-phase during quenching.

**Figure 5:**
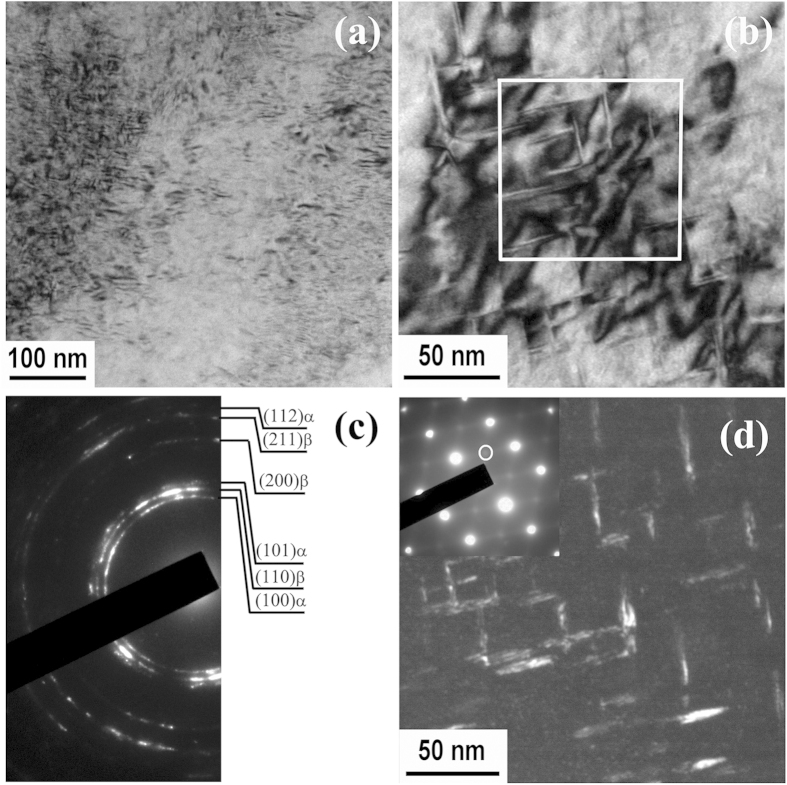
TEM images of a CRA Ti-33Nb-4Sn specimen: (a) low-magnification image showing dislocation tangles and tiny precipitates; (b) high-magnification image showing the lenticular precipitates; (c) SAD pattern of Fig. 5(a), showing diffractions from β and α-phases, consistent with XRD results (Fig. 1(b)); dark-field image with the diffraction intensity from the α-phase, showing the morphology and dimension of the α-phase precipitates.

On the other hand, the bright-field TEM image of the CRA specimen shows apparent contrast presumably caused by dislocation tangles, as shown in Fig. 5a. These dislocation tangles can be ascribed to the severe cold rolling prior to annealing. At higher magnification, fine precipitates can be identified accompanying the areas of dislocation tangles, as shown in Fig. 5b. Figure 5c shows the SAD pattern corresponding to Fig. 5a. Indexing of the SAD pattern clearly demonstrates that these fine precipitates are the α-phase, consistent with XRD results. In addition, the nearly-continuous diffraction rings in Fig. 5c suggest that the size of the grains of the CRA specimen is very small. Recent studies by the present authors have confirmed that severe cold rolling and low-temperature annealing can lead to significant grain refinement in metastable β-type Ti-Nb-based alloys^18^. Therefore, we can reasonably attribute the grain refinement in the CRA Ti-33Nb-4Sn specimen to the severe cold rolling and subsequent annealing treatment.

In order to clearly reveal the morphology and size of the α-precipitates, we performed dark-field TEM on the CRA specimen. Figure 5d shows a dark field image recorded using the encircled diffraction beam from the α-phase. It can be seen that α-precipitates exhibit lenticular morphology and are very small in size, with several nanometers in width and dozens of nanometers in length. As is well known, α-phase always preferentially nucleates at defects in β-matrix, such as dislocations, intermetallic particles and grain boundaries^23^. Since high density of dislocations and grain boundaries are present in the CRA Ti-33Nb-4Sn specimen (Fig. 5a,c), they can provide abundant heterogeneous nucleation sites for α-precipitates, promoting the precipitation of nanometer-scale α-phase particles during short-time annealing. These nanometer-scaled α-precipitates can serve as strengthening medium in the β-matrix by blocking dislocations either in the sense of Orowan mechanism (dislocation bowing out for very strong particles)^24^ or the Friedel mechanism (dislocation shearing through, when the particles are discretely distributed and relatively soft)^25^. Therefore, our detailed TEM analysis explains why the CRA Ti-33Nb-4Sn alloy exhibits high yield strength and ultimate tensile strength, as shown in Fig. 3.

**Figure 6:**
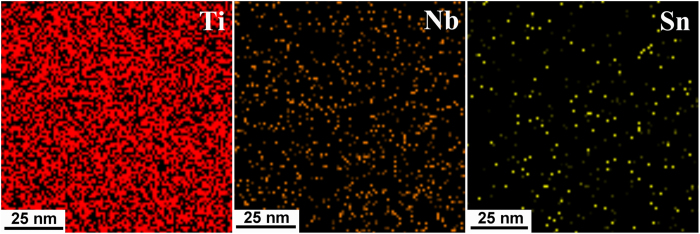
Energy dispersive spectroscopy (EDS) elemental mapping results for Ti, Nb and Sn corresponding to the boxed area covering both the β- and α-phases. No significant enrichment or depletion of these three major elements can be observed, indicating that short annealing in the CRA specimen does not considerably modify the chemical composition of the β-matrix.

In β-type Ti alloys, formation of α-phase during prolonged high-temperature annealing tends to cause enrichment of β-stabilizers in β-matrix^22^,^26^. As a result, the elastic modulus of the β-phase will increase after annealing due to the increased content of β-stabilizers in the residual β-matrix. In order to elucidate the influence of the precipitation of fine α-particles on the content of β-stabilizers in residual β-matrix, we conducted energy dispersive spectroscopy (EDS) on the CRA specimen. The EDS elemental mapping results for Ti, Nb and Sn corresponding to the boxed area covering β- and α-phases (Fig. 5) are presented in Fig. 6. Interestingly, no obvious compositional changes can be observed through EDS elemental mapping, suggesting that formation of the α-precipitates did not cause significant compositional change in β-matrix. This evidently substantiates the notion that during short-time annealing, no compositional partitioning for β-stabilizers from α-phase to β-matrix takes place. A similar result was reported in Ti-5Al-5Mo-5V-3Cr-0.5Fe β-titanium alloy by Nag *et al.*^23^. They claimed that during low-temperature or short-time annealing, initially small-sized α-precipitates form by displacive transformation instead of diffusional mechanism.

Here, we need to point out that although the precipitation of α-phase does not cause detectible increase of β-stabilizers in residual β-matrix, the martensitic transformation per se is retarded to below room temperature after thermo-mechanical treatment (Figs 1 and 2). As a matter of fact, in addition to chemical composition, microstructure can have a substantial impact on hindering martensitic transformation because of its shear-like feature^16^,^17^,^18^. In the case of Ti-33Nb-4Sn alloy, high density of dislocations and grain boundaries in the CRA specimen may be held responsible for retarding the shearing process associated with the martensitic transformation which in turn lowers the Ms temperature. As a consequence, the β-matrix with relatively low content of β-stabilizers (identical to that of ST β-phase) is retained at room temperature. We believe this provides a plausible explanation that the CRA specimen exhibits a Young’s modulus of 36 GPa due to the ultralow modulus of β matrix caused by low β-stabilizers content.

In this study, a metastable β-type Ti alloy with high ultimate strength (~853 MPa) and ultralow elastic modulus (~36 GPa versus ~30 GPa for human bone) was fabricated. In this metastable Ti alloy, β-phase with low content of β-stabilizers is retained at room temperature by high density of dislocations and grain boundaries introduced by cold rolling and annealing treatment. It is this approach and the microstructure that render the bone-compatible low Young’s modulus. Meanwhile, nanometer-sized α-precipitates serve to strengthen the β-matrix acting as dislocation barriers. We believe that this approach could be applied to the development of metastable β-type titanium alloys with ultralow Young’s modulus and high strength. In a broader context, our effort may open a new avenue for design and fabrication of novel metastable β-type titanium alloys for implant applications.

## Methods

Ingots with nominal compositions of Ti-32Nb-4Sn, Ti-33Nb-4Sn and Ti-34Nb-4Sn in mass% were fabricated by arc melting in an argon atmosphere using nontoxic high purity Ti (99.99%), Nb (99.95%) and Sn (99.95%). These ingots were re-melted four times and then homogenized at 1273 K for 4 hours in vacuum. The homogenized ingots were forged at 1173 K into the following dimension: 90 mm (length) × 50 mm (width) × 8 mm (thick). After forging, the billets were solution treated at 1073 K for 1 hour in an evacuated quartz tube, followed by quenching into water (~298 K) by breaking the quartz tube. The specimens which were cut from the solution treated billets will be referred to as ST specimens hereafter. The ST billets were cold rolled to 7.8 mm, 7.3 mm, 6.2 mm, 5 mm, 3.5 mm, 1.5 mm, and then to a final thickness of ~1 mm without intermediate annealing, with a thickness reduction of 87%. They were then annealed at 673 K for 20 minutes followed by quenching into water. These specimens will be subsequently denoted as CRA specimens hereafter. The phase constitutions of ST and CRA Ti-Nb-Sn alloys determined by X-ray diffraction analysis are shown in Table S1. Note that after the cold rolling plus annealing treatment, the critical content of Nb, where the β phase can survive against the α″ martensitic transformation, is 33% in mass. Therefore, the Ti-33Nb-4Sn alloy was chosen as the model alloy for the present study. The ST and CRA Ti-33Nb-4Sn specimens were analyzed by wet chemical analysis to characterize the differences between nominal and actual compositions, and the results are shown in Table S2. Interstitial contents of N, H and O of the cold rolled and CRA Ti-33Nb-4Sn alloys were also listed in Table S2. Since changes between nominal compositions and actual compositions were negligible in Ti-33Nb-4Sn alloys, the alloy compositions will be denoted hereafter by nominal compositions. The chemical composition of local area of the CRA Ti-33Nb-4Sn specimen was estimated using a JSM 6300 scanning electron microscope equipped with a standard energy dispersive X-ray analysis system at 30 kV, showing a homogeneous chemical composition distribution (see Fig. S4).

Phase constitution was determined by X-ray diffraction (XRD) Cu Kα irradiation at an accelerating voltage of 40 kV and a current of 200 mA. Uniaxial tensile test was conducted on an Instron-8801 testing system at a strain rate of 1 × 10^−3^ s^−1^. Tensile specimens have a rectangular cross-section of 1 × 1.46 mm^2^ and a gage length of 30 mm, with the rolling direction parallel to the loading axis. An extensometer was used for accurate strain measurement. Young’s modulus, yield strength and ultimate tensile strength were determined from the stress-strain curves. Experimental uncertainty for tensile Young’s modulus and tensile strength are ±2 GPa and ±10 MPa, respectively. In addition to the tensile elastic modulus, the dynamic Young’s modulus of the ST and CRA Ti-33Nb-4Sn specimens were also determined by a free resonance vibration technique with an experimental error of ±1 GPa, using specimens with dimensions of 60 mm × 10 mm × 2 mm from another alloy batch (Fig. S1). A dynamic mechanical analyzer (TA Q800 DMA) was employed to measure the storage modulus as a function of temperature, in a single cantilever mode with amplitude of 15 μm at dynamic stress frequency of 1 Hz and cooling rate of 5 K min^−1^. The martensitic transformation starting (Ms) temperature of the specimen was determined from the recorded storage modulus-temperature curve. Microstructural and compositional analyses were performed on a JEM 2100F transmission electron microscope (TEM) equipped with energy dispersive X-ray spectroscopy (EDS) operated at 200 kV.

## Additional Information

**How to cite this article**: Guo, S. *et al.* Design and fabrication of a metastable β-type titanium alloy with ultralow elastic modulus and high strength. *Sci. Rep.* **5**, 14688; doi: 10.1038/srep14688 (2015).

## Supplementary Material

Supplementary Information
