# Supplementary material for: Design and fabrication of a metastable β-type titanium alloy with ultralow elastic modulus and high strength
Source: Sci Rep. 2015 Oct 5;5:14688. doi: 10.1038/srep14688 (PMC4592961; doi:10.1038/srep14688)
Supplement: Supplementary Information [file srep14688-s1.pdf]

Supplementary Information

Design and fabrication of a metastable β-type titanium alloy with ultralow elastic modulus and high strength

Shun Guo[1,2]*, Qingkun Meng[1], Xinqing Zhao[1]*, Qiuming Wei[3]* & Huibin Xu[1]

[1]School of Materials Science and Engineering, Beihang University, Beijing 100191, P. R. China

[2]Institute for Advanced Materials, Jiangsu University, Zhenjiang 212013, P. R. China

[3]Department of Mechanical Engineering and Engineering Science, University of North Carolina at Charlotte, 9201 University City Blvd., Charlotte 7 NC 28223-0001, USA

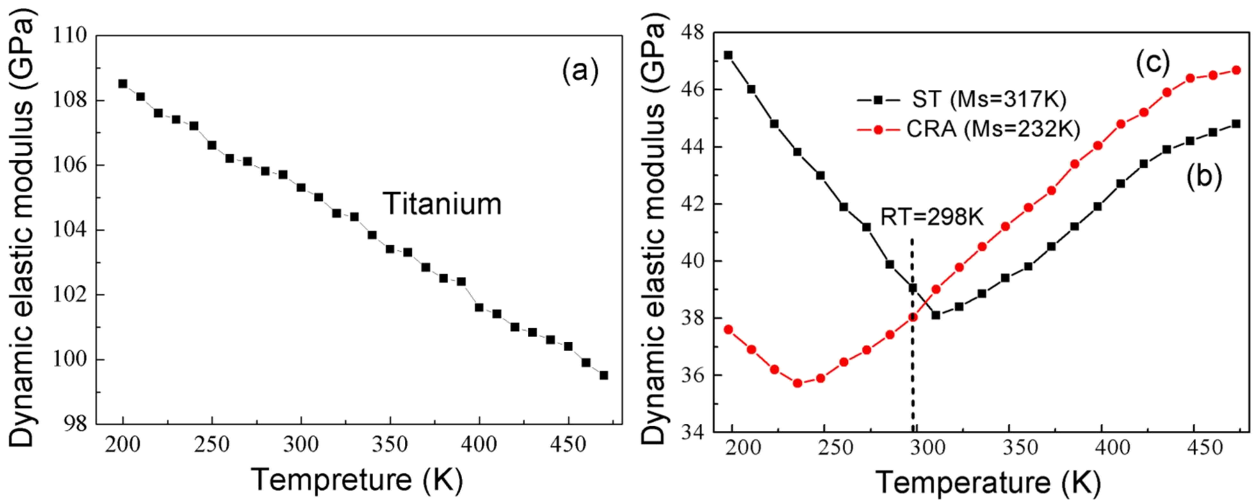

Fig. S1 Dynamic modulus versus temperature during cooling for (a) pure titanium, (b) solution treated (ST) and (c) cold rolled plus annealed (CRA) Ti-33Nb-4Sn specimens.

In order to uncover the mechanisms responsible for the ultralow elastic modulus of Ti-33Nb-4Sn alloy, we measured the dynamic Young's moduli of both ST and CRA specimens during cooling from 473 K to 198 K by free resonance vibration. The dynamic Young's modulus of pure Ti was measured under the same experimental conditions. Dimensions of the specimens are 60

mm×10 mm×2 mm. In the experiment of free resonance vibration, the intrinsic resonance frequency was measured and the dynamic Young's modulus was derived by

$$E = 0.9694\frac{\rho L^4 f_r^2}{d^2},$$

where ρ, L and d are the density, length, and thickness of specimens, respectively; f_r is the intrinsic resonance frequency. Fig. S1 shows the results for pure titanium (a), ST (b) and CRA (c) specimens of Ti-33Nb-4Sn, respectively. It is clearly shown that the dynamic Young's modulus of pure Ti increases monotonically with decreasing temperature. This can be easily explained by the temperature dependence of the stability of α-phase of pure Ti[1]. Pure Ti is known to exhibit an allotropic transition (β↔α) at 1155 K. Lowering temperature from this critical point will intensify the stability of α-phase and strengthen the bonding force of Ti atoms, thus resulting in an increased Young's modulus.

The XRD results in the manuscript (see Fig. 1) clearly suggest that the α"-martensite in ST Ti-33Nb-4Sn alloy transforms reversibly to parent β phase at 473 K. This means that at the beginning of the measurement of dynamic Young's modulus at 473 K, the ST specimen consists of β-phase only. Fig. S1 shows that the Young's modulus of ST specimen decreases with decreasing temperature down to ~312.5 K (close to the Ms of ~317 K) and then increases with further decrease in temperature. In light of the stability change of β-phase and α"-martensite with lowering temperature, we could explicate the temperature dependence of dynamic Young's moduli for ST and CRA specimens. Above Ms, lowering temperature will lead to decreasing stability of β-phase; below Ms, further lowering temperature will in turn stabilize the α"-martensite, resulting in the lowest lattice stability at around Ms. This lowest lattice stability corresponds to a minimum value of elastic modulus. Here, we believe that these interesting phenomena are closely related to the nature of thermoelastic martensitic transformation of the metastable β-Ti alloy. As a matter of fact,

softening of elastic constant (C′ or C_{44}) has been observed in other alloys where thermoelastic martensitic transformation is anticipated approaching Ms, such as TiNi and Cu-based shape memory alloys[2]. Accordingly, we may conclude that if the martensitic transformation of the metastable β-phase of a Ti-alloy can be retarded to lower temperature by thermo-mechanical treatment, a lower Young's modulus may ensue. In this context, we can reasonably understand the lower Young's modulus of the CRA specimens versus the ST specimens.

Fig. S1 indicates that at 317 K (the Ms of ST), the CRA specimen exhibits slightly higher Young's modulus than the ST specimen. This can be attributed to the presence of α precipitates in the CRA specimen, as discussed in the manuscript (Fig. 1). Note that grain refinement and dislocations induced by thermo-mechanical treatment cannot exert substantial effect on Young's modulus[3]. It is yet interesting to observe that the CRA specimens show decreasing Young's modulus with lowering temperature, achieving a minimum value (35.5 GPa) at ~237.5 K (near the Ms, 232 K), and a Young's modulus of ~38 GPa at room temperature (298 K). Note that this ultralow Young's modulus, ~38 GPa, is lower than the modulus of ST specimen at the same temperature. According to TEM and EDS analysis (Fig. 5 and Fig. 6 in the manuscript), we can reasonably attribute the delay of martensitic transformation of β-phase with low stability to grain refinement and high-density of dislocations produced during the thermo-mechanical treatment.

It is also interesting to point out that the dynamic Young's modulus (Fig. S1) is slightly higher than the corresponding tensile Young's modulus (Fig. 3) of the CRA Ti-33Nb-4Sn. This might be ascribed to the methods and specimens (shape and size) for measurement and characterization of Young's modulus, i.e. the tensile test and the free resonance vibration technique.

The room-temperature XRD results and the tensile stress-strain curve of the cold rolled Ti-33Nb-4Sn specimen annealed at 723K for 4h are presented in Fig. S2a and 2b, respectively.

Comparing Fig. S2 with Fig 1 and Fig 3, it can be observed that raising annealing temperature and extending annealing time can promote the precipitation of α, which helps to strengthen alloy. However, over-annealing leads to a large amount of α precipitates which in turn results in an increase in elastic modulus, exacerbating the “stress shielding” problem caused by the high modulus of the implant. Clearly, in comparison to high strength, low modulus is more important for implant alloys. Therefore, in the present study, short-time annealing at low annealing temperature, i.e. annealing at 673 K for 20 minutes, was employed to limit the volume fraction of α in order to obtain ultralow Young’s modulus.

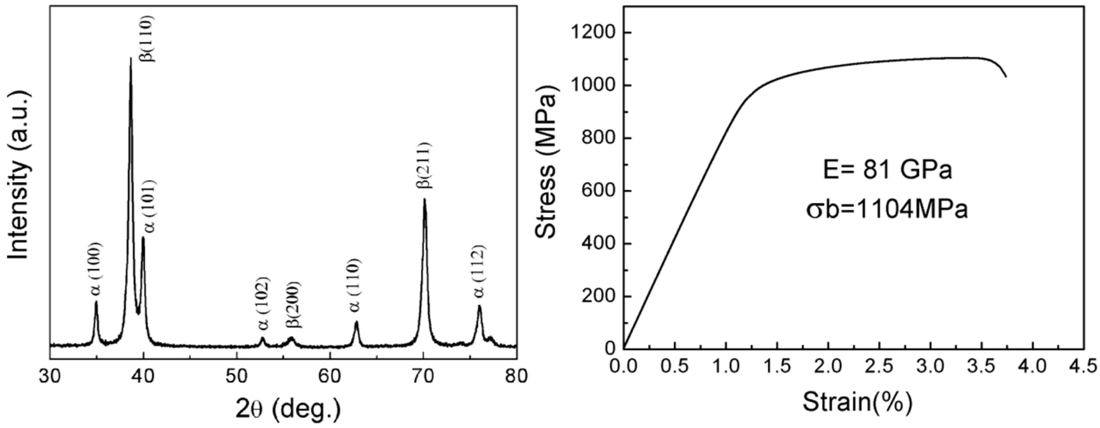

Fig. S2 The room-temperature XRD results (a) and the tensile stress-strain curve (b) of the cold rolled Ti-33Nb-4Sn specimen annealed at 723K for 4h.

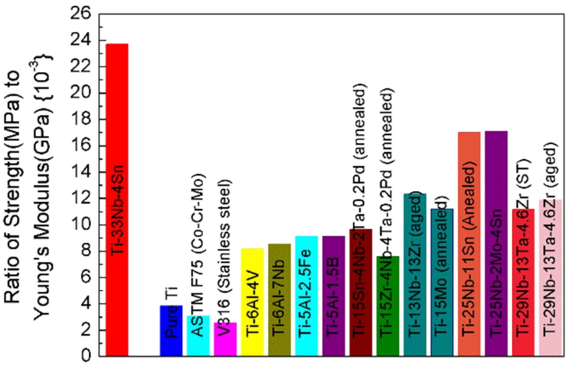

Fig. S3 The comparison of strength-to-modulus ratios of Ti-33Nb-4Sn alloy with some typical implant materials.

The strength-to-modulus ratio is often employed as the key parameter for evaluating the performance of biomaterials with low elastic modulus and high strength. The higher the strength-to-modulus ratio is, the more desirable the material for implant applications. As such, the strength-to-modulus ratio of Ti-33Nb-4Sn alloy and those of some typical implant materials are juxtaposed in Fig S3. The CRA Ti-33Nb-4Sn alloy clearly stands out with the highest strength-to-modulus ratio vis-à-vis those currently used implant materials, such as the C.P. Ti and Ti-35.3Nb-5.1Ta-7.1Zr alloy and so on[4-7]. Thus, the CRA Ti-33Nb-4Sn alloy can be a potential candidate for biomedical applications from the viewpoint of its ultralow modulus and high strength.

Table S1 Phase constitutions of ST and CRA Ti-Nb-Sn alloys.

	Ti-32Nb-4Sn	Ti-33Nb-4Sn	Ti-34Nb-4Sn
ST	α" + β	β + α"	β + (α")*
CRA	β+ (α" + α)*	β+ (α)*	β+ (α)*

* The phases, which have volume fractions below 15%, are given in the brackets.

Table S2 Chemical analysis of the cold rolled and CRA Ti-33Nb-4Sn alloys (wt%).

Specimen	Ti	Nb	Sn	O	N	H
Cold rolled	Bal.	32.8	3.93	0.064	0.0077	0.0045
CRA	Bal.	32.8	3.92	0.069	0.0080	0.0043

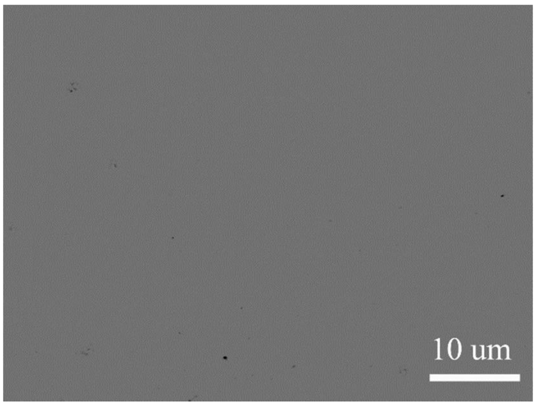

Fig. S4 Back scattered electron image of CRA Ti-33Nb-4Sn alloy.

References:

1. Liu, C. T. & Ma, J. *Mechanical properties of materials* (Chemical Industry Press, Beijing, 2009).

2. Otsuka, K., & Ren, X. B. Physical metallurgy of Ti-Ni based shape memory alloys. *Prog. Mater. Sci.* **50**, 511-678 (2005).

3. Hao, Y. L. et al. Aging response of the Young's modulus and mechanical properties of Ti-29Nb-13Ta-4.6Zr for biomedical applications. *Metall. Mater. Trans A* **34**, 1007-1012 (2003).

4. Geetha, M., Singh, A. K., Asokamani, R. & Gogia, A. K. Ti based biomaterials, the ultimate choice for orthopaedic implants - A review. *Prog. Mater. Sci.* **54**, 397-425 (2009).

5. Banerjee, D. & Williams J. C. Perspectives on Titanium Science and Technology. *Acta Mater.* **61**, 844-879 (2013).

6. Guo, S., Meng, Q. K., Cheng, X. N. & Zhao, X. Q. Deformation behavior of metastable β-type Ti-25Nb-2Mo-4Sn alloy for biomedical applications. *J. Mech. Behav. Biomed. Mater.* **38**, 26-32 (2014).

7. Jung, T. K., Semboshi, S., Masahashi, N. & Hanada, S. Mechanical properties and microstructures of β Ti-25Nb-11Sn ternary alloy for biomedical applications. *Mater. Sci. Eng. C* **33**, 1629-1635 (2013).
